# Measuring the dynamic wind load acting on standing trees in the field without destroying them

**DOI:** 10.1371/journal.pone.0323532

**Published:** 2025-05-20

**Authors:** Satoru Suzuki, Ayana Miyashita

**Affiliations:** Center for Forest Damage and Risk Management, Forestry and Forest Products Research Institute, Tsukuba, Ibaraki, Japan; King Fahd University of Petroleum & Minerals, SAUDI ARABIA

## Abstract

Wind loads are a factor in tree growth, tree architecture, and the occurrence of disasters and forest disturbances, e.g., tree falls. To understand forest ecosystems and manage forests effectively, it is necessary to understand the relationship between wind loads and trees. However, wind speed and direction always vary, which makes it difficult to measure wind loads acting on trees dynamically. We have proposed a method to accurately measure the dynamic wind load (*L*_*w*_), the centroid of the dynamic wind load distribution (*C*_*L*_), and the dynamic wind load direction (*D*_*L*_) using multiple strain gauges attached to a trunk. The advantage of this method is that it can quantify the moment by separating it into *L*_*w*_ and *C*_*L*_. However, this method was only validated in a laboratory conditions by applying static loads to a cylinder pole and a small sapling. If this method can be applied to forest environments, it should provide meaningful results in areas such as forest ecology and forest conservation. Thus, in this study, the accuracy of measurement of these values was investigated under natural wind conditions to validate the feasibility of using the proposed method in a real-world field environment. At relatively higher wind speed, the accuracy of *L*_*w*_ was less than 10% of the systematic errors and the mean absolute percentage error (MAPE), the accuracy of *C*_*L*_ was less than 7.7% of the MAPE, and the accuracy of *D*_*L*_ was 12.3° of the mean absolute error (MAE). The influences of wind turbulence, the deformation of tree crown were also investigated. The results show that fluctuations in wind speed, wind direction, and the deformation have little effect on the accuracy of the values. The method employed in this study had sufficient characteristics to measure taller standing trees than the current sample in terms of sampling frequency. Thus, the method employed in this study can be widely used to measure dynamic *L*_*w*_, *C*_*L*_, and *D*_*L*_ of standing trees with the above accuracy in real-world field conditions.

## Introduction

Both tree growth and survival are strongly related to wind loads; thus, it is necessary to measure the wind loads acting on trees to understand the morphogenesis of trees and forest ecosystems. For example, the ratio of trunk elongation to radial growth changes with loading. When trees are exposed to high winds or grown under forceful conditions that mimic high winds, elongation growth is suppressed, and hypertrophic growth is promoted [1]. Conversely, the opposite effect is observed when the trunk is fixed and allowed to grow in the absence of any force [2]. When the wind environment is regulated in wind tunnels and seedlings are grown, the root system growth is also affected. Lateral roots on the upwind and downwind sides, where the wind forces are primarily in effect, are thicker and longer [3]. Thus, both the aboveground and belowground parts of the tree grow in response to the wind loads to which they are exposed. The proposed principle for the effect of wind loading on growth is that each part of the trunk becomes enlarged, and the stress distribution in the trunk due to wind loading takes a particular form [4–6]. This hypothesis is convenient to relate trunk diameter to crown shape; however, it has not been fully verified as a mechanism for trunk formation [7], in part because wind loads cannot be measured directly.

In addition, wind loads are difficult to measure; thus, the magnitude of the wind loads and the mechanisms by which trees respond to wind loads are analyzed using both static and dynamic analysis techniques (sometimes in combination). The dynamic behavior of trees and the occurrence of damage are often assessed by estimating the moments acting on trees. For example, tree uprooting occurs when the moment applied by the wind load exceeds the strength of the root system, and snapping occurs when the stress on the trunk section exceeds the bending strength [8–10]. Several mechanical models, e.g., HWIND [11], GALES [12] and FOREOLE [13] have been developed based on this relationship. Moment is the product of the magnitude of the wind loads and the centroid of the wind load distribution. Therefore, by separately measuring the wind load and its centroid, it is possible to gain a deeper understanding of the physical phenomenon and to accurately estimate the moment.

In a previous study we proposed a method to measure the magnitude and direction of the wind load and the centroid of the wind load distribution acting on the tree using four strain gauges attached to the trunk of the tree[14]. The characteristics of this method are that it is possible to measure the moment by separating it into wind loads and centroid of wind loads, and that these can be measured with very high accuracy. However, the validity of the method was tested only for the samples subjected to static loading. To apply this method to forest trees, the measurement accuracy is required to be tested in natural wind environments where wind speed and direction vary over time.

In this study, our goal is to verify the accuracy of the proposed method regarding wind speed and the variation of wind speed/direction and to test the possibility of applying this method under field conditions. For this, we conducted the measurements of the magnitude, direction, and centroid of the wind load distribution acting on young Japanese ceder trees under natural wind conditions.

## Materials and methods

### Experimental design and apparatus

**Fig 1:**
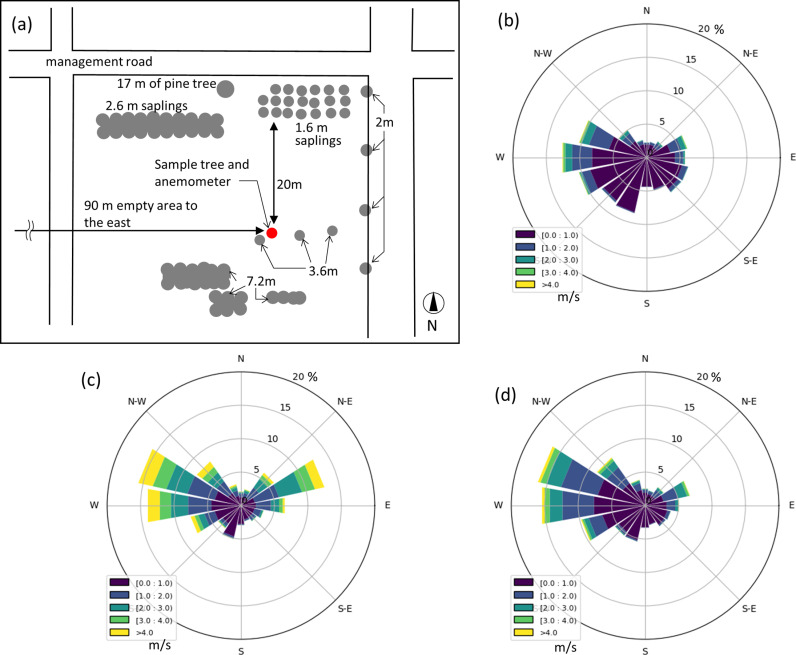
The arrangement of measurement location and surrounding trees (a), and the percentage of occurrences of wind speed and direction during the measurement period for Tree 1 (b), Tree 2 (c), and Tree 3 (d).

The study was conducted at the experimental field of the Forestry and Forest Products Research Institute (36°00′25″N, 140°7′37″E). The main wind direction at the study site was west, and there was no obstruction in the range of 20–90 m on a bearing from north to west, including the primary wind direction (Fig 1).

**Fig 2:**
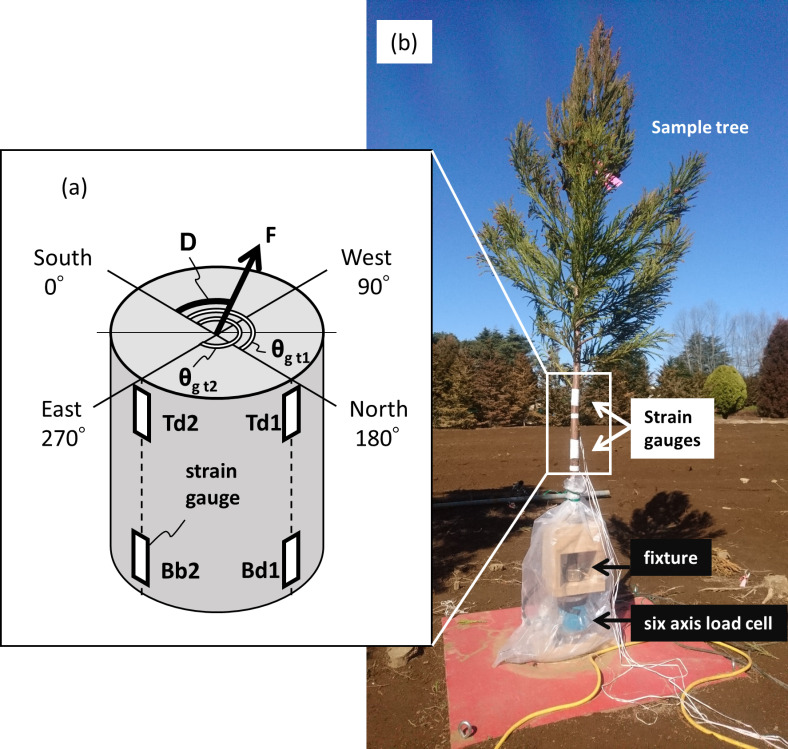
Diagrams of the arrangement of strain gauges and the experimental apparatus. Schema of the arrangement of strain gauges (a), fixing of sample tree (Tree 2) to load cell and arrangement of sensors (b) are shown.

**Fig 3:**
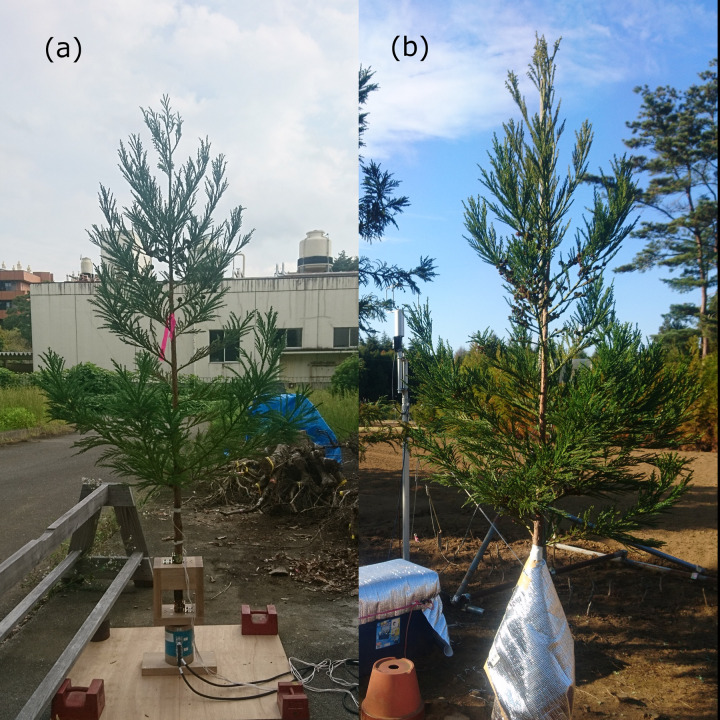
Photographs of the sample trees. Tree 1 (a) and Tree 3 (b) are shown. Tree 2 was shown in Fig 2b.

A 2–3 m section was cut from the top end of each of the sample trees, and four strain gauges were attached to the lower part of each trunk and placed on a six-axis load cell (LMC-61281, Nissho-Electric-Works, Tokyo, Japan) via a fixture (Fig 2). Three cedar trees (*Cryptomeria japonica*) were used for the measurements (Fig 3 and Table 1). The six-axis load cell measures the loads and moments in the three orthogonal axes acting on the target object. To avoid exceeding the measurement limits of each component of moment, the height of the sample trees was limited to less than 3 m.

**Table 1:** Sample trees.

Tree No.	Height (cm)	Center of gravity (cm)	Mass (kg)	Projected area^*^ (m^2^)	Diameter at gauges (cm)		Height at gauges (cm)		Natural Frequency (Hz)
					T	B	T	B	
**1**	205	89	2.4	0.84	3.0	3.2	45	32	1.5
**2**	233	119	3.3	0.70	3.1	3.4	67	47	0.9
**3**	286	131	4.9	1.34	3.4	3.7	65	41	0.8

*Projected area is the tree crown area projected onto a vertical plane.

Strain gauges (KFRP-5-120-C1-1L5M3R, Kyowa Electronic, Tokyo, Japan) were attached using cyanoacrylate adhesive after removing the bark and the cambium layer from the trees. The strain gauges were attached to nearly orthogonal surfaces on the north and east sides of the trunks (the east and south sides for Tree 1). The strain gauge and six axis load cell readings were recorded at 10 Hz using a data logger (EDX-2000A, Kyowa Electronic, Tokyo, Japan). The measurement period for each sample tree was approximately one month. Note that this examination was conducted in winter, and cedar is relatively drought tolerant; thus the leaves remained green during the experiment period and no apparent changes were observed. The ultrasonic anemometer was fixed 3 m west of the sample trees at a height of 3 m. The three wind speed components were measured using an ultrasonic anemometer (CYG-81000, R.M. Young, MI, USA) and recorded on a data logger (CR3000X, Campbell, UT, USA) at 10 Hz.

Data from four days with high winds were analyzed (Table 2), and the analysis did not include the range where *L*_*W*_ was small due to low wind speed, i.e., *L*_*W*_ < 1.0 N and *L*_*w_ref*_ < 1.0 N. Accuracy was verified by the mean absolute error (MAE) and mean absolute percentage error (MAPE) against the six-axis load cell readings. The natural frequencies of each sample tree were obtained from FFT analysis of the free vibration waveforms.

**Table 2:** Meteorological conditions on the day used for analysis. Data from the four days with the strongest wind speed during the measurement period were analysed. The measurements were taken at a height of 3m above the ground.

Tree No.	Date	Max. 10 min mean wind speed (m s^-1^)	Max. wind speed measured at 10 Hz (m s^-1^)	Daily mean air temperature ( °C )
**1**	Oct. 20^*^	1.9	6.2	14.5
	Oct. 31	1.5	5.6	9.5
	Nov. 2	1.6	4.5	13.8
	Nov. 4	2.8	9.8	10.2
**2**	Mar. 6	2.5	8.0	11.9
	Mar. 10	4.2	13	9.3
	Mar. 13	3.8	11.6	11.1
	Mar. 14	4.6	15.7	11.1
**3**	Dec. 12	2.0	6.7	9.6
	Dec. 13	2.4	7.7	7.1
	Dec. 19	2.6	8.3	2.4
	Dec. 20	3.3	11.3	2.2

*data is available for half a day.

### Calculating *L*_*W*_, *C*_*L*_ and *D*_*L*_

The magnitudes of the wind load (*L*_*W*_), the height of the centroid of the distributed load (*C*_*L*_), and the direction of the wind load (*D*_*L*_) acting on the trees were calculated using a formulation of the technique presented by Miyashita and Suzuki [14]:


$$\begin{array}{c} L_{W}=\frac{E_{B}\varepsilon_{B}Z_{B}-E_{T}\varepsilon_{T}Z_{T}}{\left(h_{T}-h_{B}\right)}, \end{array}$$ (1)

$$C_{L}=\frac{E_{T}\varepsilon_{T}Z_{T}\left(h_{T}-h_{B}\right)}{E_{B}\varepsilon_{B}Z_{B}-E_{T}\varepsilon_{T}Z_{T}}+h_{T},\;$$ (2)

$${\;D_{L}|}_{T,B}={\;arctan\left(\frac{{\varepsilon_{d1}E}_{d1}Z_{d1}\cos\theta_{gd2}-\varepsilon_{d2}E_{d2}Z_{d2}\cos\theta_{gd1}}{{\varepsilon_{d2}E}_{d2}Z_{d2}\sin\theta_{gd1}-{\varepsilon_{d1}E}_{d1}Z_{d1}\sin\theta_{gd2}}\right)|}_{T,B.}$$ (3)

Here, ε, E, Z and h are the measured strain, Young’s modulus, section modulus and height of the attached gauge, respectively, and the subscripts T and B indicate the upper and lower trunk locations, respectively. In addition, the subscripts d1 and d2 are the orientation on the attached trunk circumference (Fig 2). There are d1 and d2 strain gauges at each of the heights of T and B; thus, the direction of the wind load has two types of values calculated at T and B (D_L_ | _T_ and D_L_ | _B_). In this study, D_L_ was obtained as the vector direction that is a composite of the unit vectors in the D_L_ | _T_ and D_L_ | _B_ directions. Here, E_d1_, E_d2_, θ_gd1_, and θ_gd2_ are the values obtained by a pulling test. Note that Z_d1_ | _T_ = Z_d2_ | _T_ and

Z_d1_ | _B_ = Z_d2_ | _B_ were assumed in this study. A low-cut filter with a cutoff frequency of 0.0083 Hz (period of 120 s) was applied to ε_T_ and ε_B_. To identify E and θ in Equation 1–3, pulling tests were performed during the measurement period for each sample tree (S1 File).

### Obtaining the reference value using a six-axis load cell

The reference values for the wind load magnitude (*L*_*w _ref*_), the centroid of the wind load distribution (*C*_*L_ref*_), and the wind load direction (*D*_*L_ref*_) were calculated using Equations (4–6) with a six-axis load cell:


$$\begin{array}{c} L_{w\_ref}=\;\sqrt{{F_{x}}^{2}+\;{F_{y}}^{2}} \end{array}$$ (4)

$$\begin{array}{c} C_{L\_ref}=\;\frac{\sqrt{{M_{x}}^{2}\;+\;{M_{y}}^{2}}}{L_{w\_ref}},\; \end{array}$$ (5)

$$D_{L\_ref}=\;\arctan\left(\frac{F_{y}}{F_{x}}\right),\;$$ (6)

where *F*_*x*_, *F*_*y*_ and *M*_*x*_, and *M*_*y*_ are the loads and moments in two orthogonal directions in the horizontal plane, respectively. A low-cut filter with a cutoff frequency of 0.0083 Hz (period of 120 s) was applied to *F*_*x*_, and *F*_*y*_.

### Identifying wind fluctuation and its properties

The degree of wind speed fluctuation was quantified by the turbulence intensity shown in Equation 7:


$$I=\frac{\sigma_{U}}{U_{m}}\;$$ (7)

where *U*_*m*_ and σ_*U*_ (m/s) are the mean and standard deviation of the wind speed for each 3 s period, respectively.

In addition to the magnitude of the wind direction, variability was quantified using a method defined by Yamartino [15], which is expressed as follows:


$$\sigma_{WD}=\;arcsin(\delta )\left[1+0.1547\delta^{3}\right]\;.$$ (8)

Here,

$\sigma_{WD}$ is the standard deviation over 3 s of the wind direction measured at 10 Hz, and δ is obtained as follows:


$$\delta =\sqrt{1-\frac{1}{N^{2}}\left[{\left\{\sum_{i}\left(\frac{U_{i\_x}}{U_{i}}\right)\right\}}^{2}+{\left\{\sum_{i}\left(\frac{U_{i\_y}}{U_{i}}\right)\right\}}^{2}\right]}.$$ (9)

Here, *N* is the number of data points, and *U*_*i_x*_ and *U*_*i_y*_ are the orthogonal wind velocity components of *U*_*i*_ measured at 10 Hz.

### Calculating drag coefficients as an indicator of deformation and susceptibility to wind

Wind varies dynamically in terms of both speed and direction, and trees respond to wind loads like a smart structural system whose response varies with the magnitude of the load [16]. Typically, the magnitude of wind loads acting on trees is estimated by multiplying the square of the wind speed, the wind-receiving area, and the drag coefficient [11,17]. The drag coefficient is the factor used to convert the wind’s velocity pressure into wind load acting on trees [18,19], and it is reflected by the degree of deformation of the tree crown [16,20] and the amount and presence of leaves[17]. Therefore, considering the drag coefficient as an indicator of deformation, we examined the relationship between the drag coefficient and measurement accuracy. The drag coefficient was calculated as follows:


$$C_{d}=\frac{2L_{w}}{\rho AU^{2}}$$ (10)

$$C_{d\_ref}=\frac{2L_{w\_ref}}{\rho AU^{2}}$$ (11)

where *C*_*d*_ is the drag coefficient for sample trees, *C*_*d_ref*_ is for reference. ρ is the air density. *A* is the sample tree’s projected area on still-air (Table 1). *U* is wind speed.

## Results

### Comparison of 10-Hz measurement values with reference values

**Fig 4:**
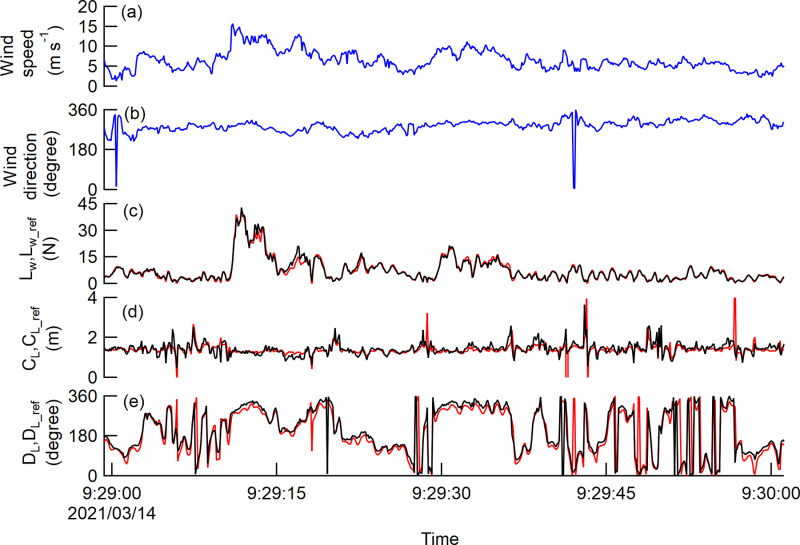
Time course of the values at 10-Hz measurement. The data were given for one minute from Tree 2, including the time at which the maximum wind speed was recorded. The time course of the wind speed (a) and the direction (b) are shown in blue lines. The red and black lines indicate the measured and reference values for graphs (c), (d), (e).

**Fig 5:**
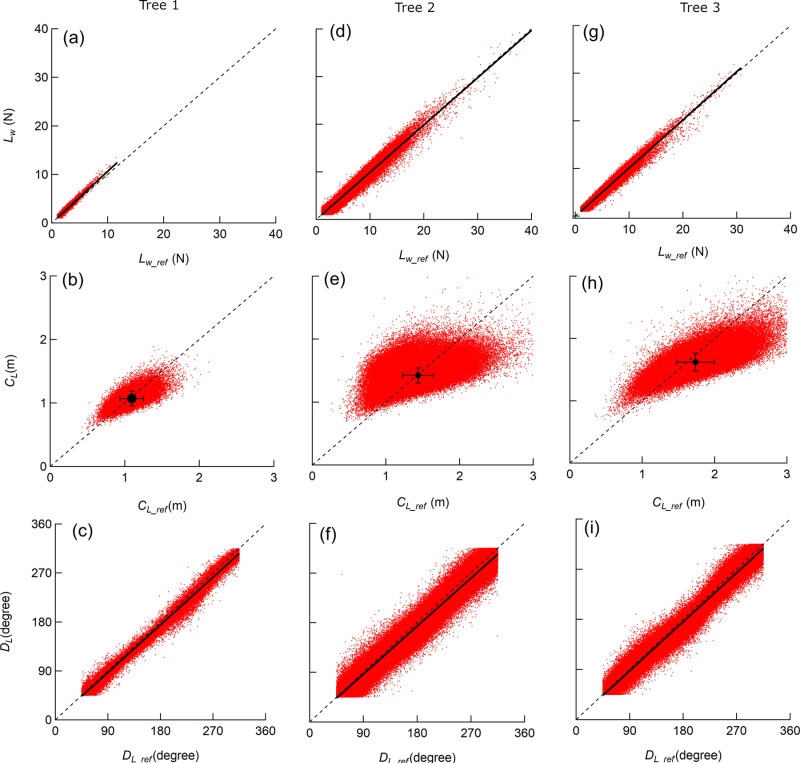
The measured values against the reference values at 10-Hz measurement. The measured and the reference values were obtained at 10 Hz for the wind load ((a), (d), (g)), the centroid of the distributed wind load ((b), (e), (h)), and the direction of the wind load ((c), (f), (i)) for each sample tree. Dashed line denotes 1:1 line. The dot and the error bars in (b), (e) and (h) denote mean value and the standard deviation.

**Fig 6:**
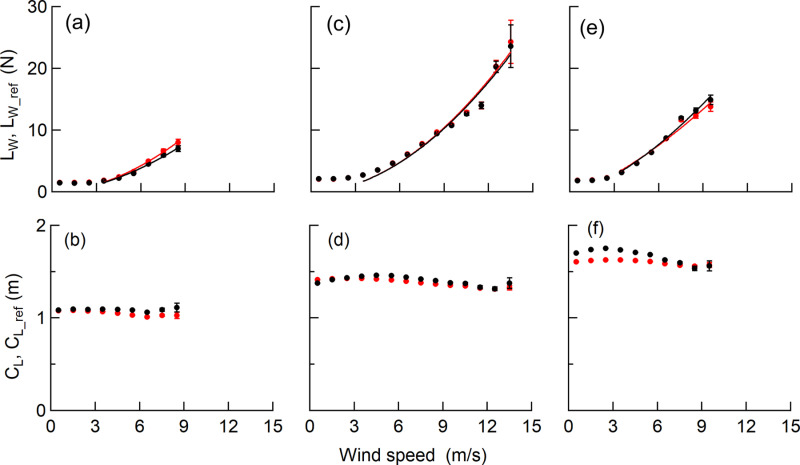
Comparison of 10-Hz measurement values for wind speed. The course of the class mean *L*_*W*_, *L*_*w_ref*_ ((a), (c), (e)) and *C*_*L*_, *C*_*L_ref*_ ((b), (d), (f)) are shown for each sample tree. These were obtained by averaging 10 Hz measurement values for wind speed classes. Error bars denote the standard error. Wind loads were regressed on a power function above 3 m/s with an intercept of zero as the analysis was carried out in *L*_*W*_, *L*_*w _ref* _> 1.0 N. Red circles and lines indicate the measured values. Black circles and lines indicate the reference values.

The time course of *L*_*w*_ and *L*_*w_ref*_ appeared to fluctuate in accordance with the variation of the wind speed (Fig 4a and c). In fact, *L*_*w*_ and *L*_*w_ref*_ increased with higher wind speed (Fig 6a, c and e) and the increasing rates of *L*_*w*_ and *L*_*w_ref*_ with wind speed were indicated with multipliers of 1.9, 1.9, and 1.4 for *L*_*W*_, and multipliers of 1.8, 1.9, and 1.5 for *L*_*w _ref*_ from Trees 1–3, respectively, when regressed on a power function. The regression coefficients of *L*_*w*_ against *L*_*w_ref*_ for each sample tree were 1.00–1.07 (Fig 5a, d and g) and the coefficients of determination were greater than 0.94 for the linear regressions with an intercept of zero (Table 3).

**Table 3:** Results related to the accuracy.

Tree No.	1	2	3	Overall
Regression coefficients of correlation between *L*_*w*_ vs *L*_*w_ref*_ (*R*^*2*^)	1.07 (0.94)	1.00 (0.96)	1.06 (0.97)	－
Regression coefficients of correlation between D_L_ vs D_L_ref_ (*R*^*2*^)	0.97 (0.98)	0.97 (0.98)	0.98 (0.98)	－
mean *C*_*L*_, mean *C*_*L*_ref_ (m)	1.07, 1.09	1.42, 1.44	1.56, 1.73	－
sd *C*_*L*_, sd *C*_*L_ref*_ (m)	0.12, 0.16	0.12, 0.21	0.12, 0.26	－
mean *C*_*L*_/H, mean *C*_*L_ref*_/H	0.51, 0.53	0.59, 0.60	0.54, 0.58	－
MAE_L_ (N)	0.18	0.30	0.27	0.28
MAPE_L_ (%)	10.8	11.5	11.6	11.5
MAE_C_ (m)	0.09	0.15	0.22	0.17
MAPE_C_ (%)	8.6	10.6	11.9	11.0
MAE_D_ (degrees)	8.6	10.4	8.6	9.6

MAE is the mean absolute error of [(1/n)Σ|(measured value)-(reference value)|]. MAPE is the mean absolute percentage error of [(100/n)Σ|{(measured value)-(reference value)}/(reference value)|]. The subscripts L, C, and D in MAE and MAPE indicate the wind load, the centoid of the distributed wind load, and the direction of the wind load. The overall values are calculated from all the sample trees. sd:standard deviation, H:tree height.

Although large amplitudes were occasionally seen for short periods of time, the time course of *C*_*L*_ and *C*_*L_ref*_ appeared to fluctuate around a certain value (Fig 4d). The fluctuation of the *C*_*L*_ and *C*_*L_ref*_ ranged from 0.8 m to the tree height. (Fig 5b, e and h). *C*_*L*_ tended to deviate from 1:1 as *C*_*L_ref*_ increased, and within the range, *C*_*L*_ was consistently smaller than *C*_*L_ref*_. The class mean *C*_*L_ref*_ of Tree 3 showed a decrease of up to 0.2 m in response to an increase in wind speed, but for others, the range of fluctuation of the class mean *C*_*L*_ and *C*_*L_ref*_ was within 0.1 m and was almost constant regardless of wind speed (Fig 6b, d and f). The class mean *C*_*L*_ and *C*_*L_ref*_ closely matched for Tree 1 and 2 (Fig 6b and d), and the residuals of the mean *C*_*L*_ and the mean *C*_*L_ref*_ were 0.02 m for both the sample trees (Table 3). For Tree 3, the residuals of the mean *C*_*L*_ and the mean *C*_*L_ref*_ were relatively larger than other sample trees, with a value of 0.17 m (Table 3), but they were in good agreement at wind speeds of 6m/s or more (Fig 6f).

The relationship between wind direction and the *D*_*L*_, *D*_*L_ref*_ in the case shown in Fig 4 indicated that the wind direction remained almost constant at 270° over time (Fig 4b), but *D*_*L*_ and *D*_*L_ref*_ fluctuated with a large amplitude (Fig 4e). In terms of *D*_*L*_ against *D*_*L_ref*_, we observed no apparent influence of the direction of the wind load as shown in the regression coefficient for each sample tree was greater than 0.97 (Fig 5c, f and i), and the coefficient of determination was greater than 0.98 for the linear regressions with an intercept of zero (Table 3).

### Influence of wind speed on MAE and MAPE values

**Fig 7:**
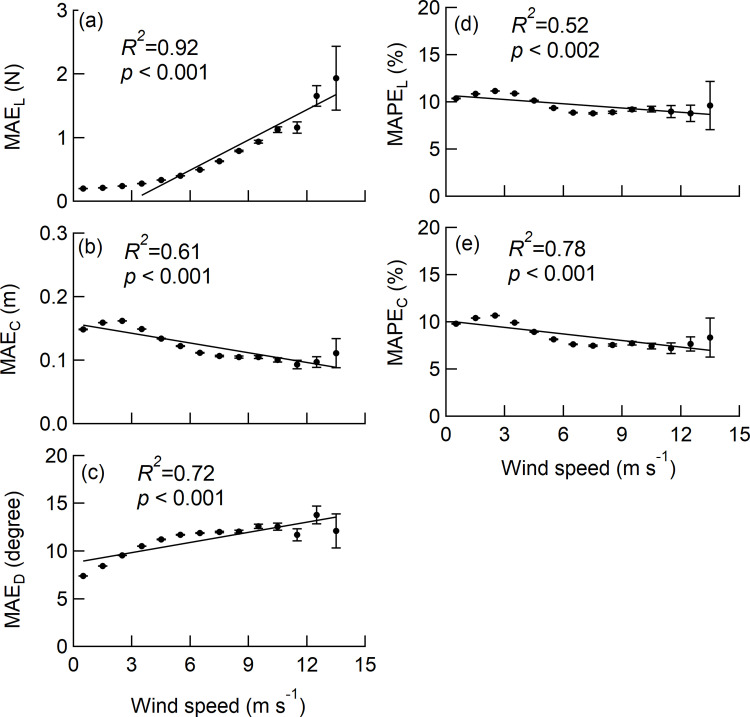
Influences of wind speed on the accuracy of *L*_*W*_,*C*_*L*_, and *D*_*L*_. The class mean values were obtained from the values measured at 10 Hz for all sample trees. Since the MAPE of *D*_*L*_ cannot be defined, only the MAE_D_ is shown for *D*_*L*_. Error bars denoted the standard error. The *R*^2^ in the linear regression and the *p* value for the test on the regression coefficients are shown in the graphs. The significance of the regression coefficients was tested using a t-test.

The MAE of *L*_*W*_ (MAE_L_) for the wind speed class increased significantly with higher wind speed (Fig 7a). In contrast, the MAPE of *L*_*W*_ (MAPE_L_) for the wind speed class indicated a significant decrease (Fig 7d). The MAE_L_ for each sample tree was 0.18–0.30 N, and the MAPE_L_ for each sample tree was 10.8–11.6% (Table 3). To examine the overall trend, we calculated the MAE_L_ and MAPE_L_ for all sample trees, and found that they were 0.28 N (SE = 0.0002 N, n = 1653456) of MAE_L_ and 11.5% (SE = 0.007%, n = 1653456) of MAPE_L_ (Table 3).

Both the MAE of *C*_*L*_ (MAE_C_) and MAPE of *C*_*L*_ (MAPE_C_) for the wind speed class decreased with increasing wind speed, and the trends of the respective regression lines were significant (Fig 7b and e). The MAE_C_ values for each sample tree were 0.09–0.33 m, and the MAPE_C_ values for each sample tree were 8.6–11.9% (Table 3). The overall MAE_C_ and MAPE_C_ values for all sample trees to investigate the overall trend were 0.17 m (SE = 0.0001 m, n = 1653456) and 11.0% (SE = 0.007%, n = 1653456), respectively (Table 3).

The MAE of *D*_*L*_ (MAE_D_) for the wind speed class increased with increasing wind speed, and the trend of the regression line was significant (Fig 7c). According to the regression line, the MAE_D_ value is expected to be 12° at the maximum wind speed class. In addition,MAE_D_ values for each sample tree were 8.6–10.4° (Table 3). The overall MAE_D_ value for all sample trees to investigate the overall trend was 9.6° (SE = 0.006°, n = 1653456) (Table 3).

### Influence of wind turbulence on *L*_*W*_,*C*_*L*_, and *D*_*L*_ measurements

**Fig 8:**
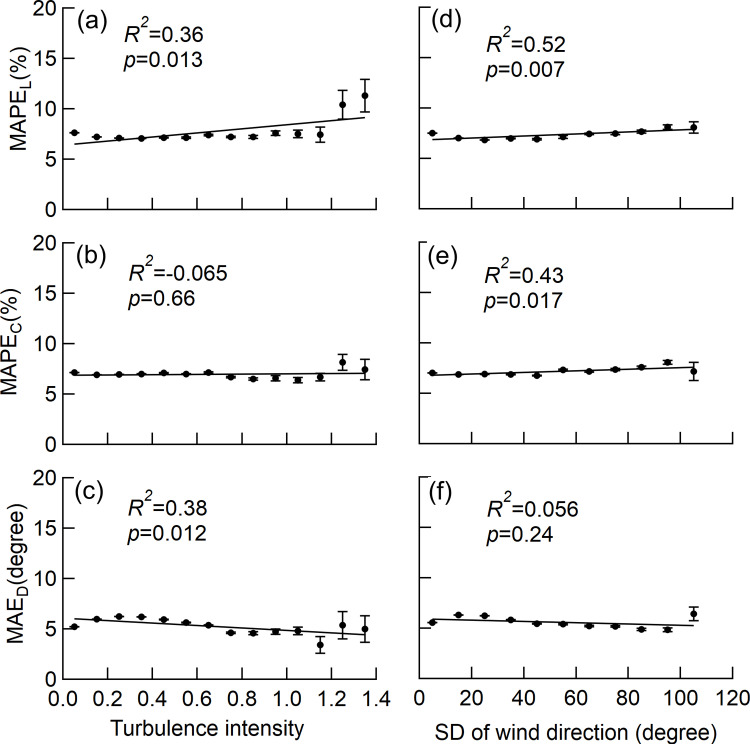
Influences of wind turbulence on the accuracy of *L*_*W*_,*C*_*L*_, and *D*_*L*_. The class mean MAPE_L_, MAPE_C_ and MAE_D_ for the turbulent intensity of the wind speed ((a)-(c)), and the standard deviation of the wind direction ((d)-(f)) were shown. The values were obtained from all the sample trees with averaging 3 seconds readings. Error bars denoted the standard error. The *R*^2^ in the linear regression and the *p* value for the test on the regression coefficients of MAPE_L_, MAPE_C_, and MAE_D_ are shown in the graphs. The significance of the regression coefficients was tested using a t-test.

The MAPE_L_ value increased with increasing turbulence intensity and the standard deviation of the wind speed, and the observed trend was significant (Fig 8a and d); however, the coefficient of determination for the regression line was a low value (*R*^*2*^ = 0.36) for turbulence intensity. The MAPE_C_ value was nearly constant over the turbulence intensity, and the trend of the regression line for MAPE_C_ was not significant (Fig 8b). The MAPE_C_ value increased slightly as the standard deviation of the wind direction increased, and the trend of the regression line for MAPE_L_ was significant (Fig 8e). The MAE_D_ value decreased slightly with increasing turbulence intensity, and the trend found to be significant (Fig 8c); however, the coefficient of determination for the regression line was low (*R*^*2*^ = 0.38). In addition, the MAE_D_ value was nearly constant over the standard deviation of the wind direction, and the trend of the regression line for MAE_D_ was not significant (Fig 8f).

### Influence of deformation on *L*_*W*_, *C*_*L*_, and *D*_*L*_ measurements

**Fig 9:**
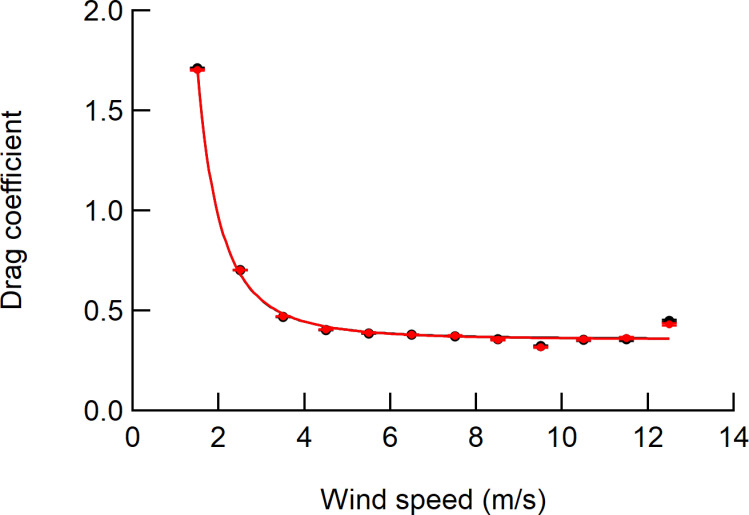
Influences of wind speed on the drag coefficient. The class mean drag coefficient for the wind speed was shown. The values were obtained by averaging 3 seconds readings. Error bars denote the standard error. Red circles and curves indicate the measured values. Black circles and curves indicate the reference values. The regression curve equation is *C*_*d *_= 0.36 + 4.14*U*^-2.77^ and *C*_*d_ref *_= 0.36 + 4.21*U*^-2.80^.

**Fig 10:**
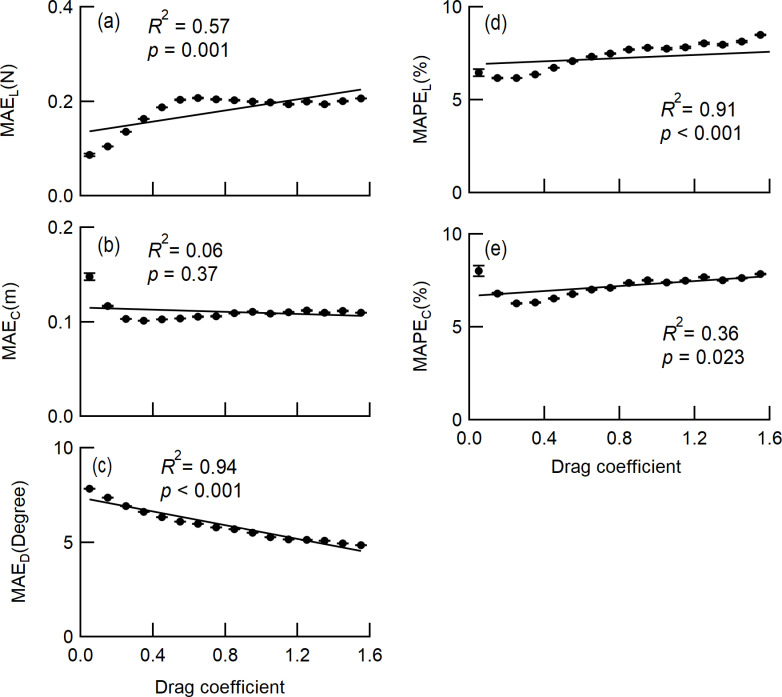
Influences of drag coefficient on the accuracy of *L*_*W*_, *C*_*L*_, and *D*_*L*_. The class mean values were obtained by averaging 3-second readings. Since the MAPE of *D*_*L*_ cannot be defined, only the MAE_D_ is shown for *D*_*L*_. Error bars denote the standard error. The *R*^2^ in the linear regression and the *p* value for the test on the regression coefficients are shown in the graphs. The significance of the regression coefficients was tested using a t-test.

The drag coefficient decreased rapidly with increasing wind speed and became almost constant above a wind speed of 5 m/s, approaching asymptotically to 0.36 for both *C*_*d*_ and *C*_*d_ref*_ (Fig 9). This means that the *C*_*d*_ value decreases because the greater the wind speed, the greater the deformation of the crown to the wind. In addition, the deformation to the wind are considered to be almost constant at wind speeds of 5 m/s or more. The *L*_*w*_ showed a significant tendency for MAE and MAPE to decrease with decreasing drag coefficient (Fig 10a and d). The MAE_C_ and MAPE_C_ had small coefficients of determination (*R*^*2*^ = 0.06 for MAE_C_, *R*^*2*^ = 0.36 for MAPE_C_) and the effect of the drag coefficient was considered to be negligible (Fig 10b and e). The MAE_D_ increased significantly with smaller drag coefficients significantly (Fig 10c).

## Discussion

### Measurement accuracy

#### Accuracy of *L*_*w*_.

As the wind speed increased, we found that *L*_*w*_ and *L*_*w_ref*_ increased rapidly, and the regression on the power function exhibited multipliers of 1.4–1.9 (Fig 6a, c and e). This indicates that the wind loads reflecting the wind pressure, which increases in proportion to the square of the wind speed, can be measured by using the method employed in the current study. As can be seen from Equations 10 and 11, as the drag coefficient decreases, the rate of increase of the wind load with respect to the increase in wind speed decreases. As the wind speed increased, the drag coefficient decreased (Fig 9), so that the multipliers were probably less than 2. The response of the drag coefficient to wind speed depends on the degree of deformation of the crown, and the multipliers likely reflect the characteristics of the response to the wind loading for each sample tree.

The results show opposite trends with wind speed: MAE_L_ increases while MAPE_L_ decreases (Fig 7a and d). This is due to the fact that the increasing rate of *L*_*w_ref*_ with respect to the increase in wind speed exceeds the increasing rate of MAE_L,_ as is evident from the MAPE_L_ formula. As the error of *L*_*w*_ decreased with increasing wind speed, MAPE_L_ improved from 11.5% of the overall value to 9.1% in the higher wind speed range (wind speed >6 m/s) (Fig 7d). Since *L*_*w*_ is calculated from the difference in strain at the upper and lower positions (Eq. 1), higher wind speeds result in greater strain differences on the trunk; thus improving the accuracy of *L*_*w*_. In addition, the accuracy of *L*_*w*_, with regression coefficients for *L*_*w*_ and *L*_*w_ref*_ of 1.00–1.07, indicates that the systematic error is within 7% (Table 3).

In terms of the wind turbulence, the accuracy of *L*_*w*_ tended to worsen with increasing turbulence intensity and the standard deviation of wind direction, as demonstrated by the significant increase in MAPE_L_ (Fis. 8a and 8d). However, the worsening of the MAPE_L_ values was marginal, with only three points deterioration for the turbulence intensity and only one point deterioration for the standard deviation of wind speed over the observed range. In terms of wind turbulence, these results indicate that wind direction and fluctuations in wind speed, and the wind direction have little effect on the accuracy of the *L*_*w*_ measurements.

In terms of the drag coefficient, the fact that MAE_L_ and MAPE_L_ decrease when drag coefficient is small indicates that accuracy improves as the deformation is greater and less susceptibility to the wind. However, since the improvement in MAE_L_ is 0.1N and the improvement in MAPE_L_ is 1 point in the range of measurement, the effect of drag coefficient to the accuracy is thought to be small.

We usually make observations for the purpose of quantifying *L*_*w*_, *C*_*L*_, and *D*_*L*_ under relatively higher wind speed conditions. Thus, it can be concluded that the *L*_*w*_ measurements can be made practically with minimal influence from wind turbulence and deformation of the tree crown under real-world field conditions, with an accuracy of less than 10% of the systematic errors and the MAPE_L_ in the range of higher wind speed.

#### Accuracy of *C*_*L*_.

The accuracy of the *C*_*L*_ tended to improve at higher wind speeds, as shown by the decrease in MAE_L_ and MAPE_L_ value (Fig 7b and e). Since *C*_*L*_ is calculated using the difference in strain at the upper and lower positions (Eq. 2), higher wind speeds result in greater strain differences on the trunk, which in turn improves the accuracy of *C*_*L*_. The improvement of accuracy was approximately 0.1 m in MAE_C_ and approximately 3 points in MAPE_C_ over the observed wind speed range (Fig 7b and e). In addition, the accuracy at higher wind speeds (wind speed > 6 m/s) remained nearly constant, with an MAE_C_ of 0.1 m and an MAPE_C_ of 7.7%.

In terms of wind turbulence, the magnitude of the fluctuations of wind speed did not influence the accuracy of *C*_*L*_ because the MAPE_C_ value did not demonstrate a significant trend (Fig 8b). The increased fluctuations in wind direction tended to slightly worsen the accuracy of *C*_*L*_, as demonstrated by the significant increase in the MAPE_C_ value for the standard deviation of the wind direction (Fig 8e). However, the deterioration of the accuracy of *C*_*L*_ for wind direction fluctuation was marginal, showing only 1 point deterioration over the range of standard deviation of the wind direction. These results indicated that wind turbulence, as demonstrated by the fluctuations in wind speed and direction, has little effect on the accuracy of *C*_*L*_ measurements.

In terms of the drag coefficient, the determination coefficients for MAE_C_ and MAPE_C_ were small, suggesting that the deformation and the susceptibility to the wind had little effect on the *C*_*L*_ measurement.

Thus, it can be concluded that *C*_*L*_ measurements could be made under real-world field conditions with an accuracy of approximately 0.17 m of MAE_C_ and 10.8% of MAPE_C_ on average (Table 3), and even better accuracy was expected at higher wind speeds with an accuracy of approximately 0.10 m of MAE_C_ and 7.7% of MAPE_C_.

#### Accuracy of *D*_*L*_.

Since *D*_*L*_ represents the direction in which the trunk has been displaced, its value depends on the position reflected by the trunk’s sway and the wind load at a given moment. The sensitivity of a particular strain gauge is at maximum when the direction of the load is 180° or 0° against the sensitive direction of the gauge, and the minimum sensitivity is 0 when the direction of the load is 90° or 270°. There was a concern that the relationship between the strain gauges and the wind load direction may appear as the directionality of the *D*_*L*_ error. However, the directionality of the error was hardly observed because the regression coefficient of *D*_*L*_ against *D*_*L_ref*_ was greater than 0.97 with *R*^*2* ^> 0.98 (Table 1). It has been reported that the accuracy was improved considerably by identifying the gauge mounting orientation and gauge sensitivity precisely by conducting pulling tests in multiple orientations [14]. The fact that relatively accurate *D*_*L*_ measurements were possible in this study regardless of the wind loading direction may be attributed to the fact that the pulling tests were performed in a similar manner.

The accuracy of *D*_*L*_ tended to worsen as the wind speed increased, as indicated by the significant increase in MAE_D_ (Fig 7c). In addition, the accuracy in the range of higher wind speed (wind speed > 6 m/s) looked nearly constant, with an MAE_D_ of 12.3°. The overall MAE_D_ for all sample trees was 9.6° at 10-Hz measurement.

In terms of wind turbulence, the accuracy of *D*_*L*_ tended to improve as the turbulence intensity of the wind speed, as demonstrated by the significant decrease in MAE_D_ (Fig 8c). However, the decrease in MAE_D_ estimated from the regression line was only 2° over the range of the turbulence intensity, and the coefficient of determination for the regression line was a low value (*R*^*2*^ = 0.36). In addition, the MAE_D_ value did not trend significantly with the standard deviation of the wind direction (Fig 8f).

In terms of the drag coefficient, MAE_D_ increased as the drag coefficient decreased. This means that the measurement accuracy of *D*_*L*_ deteriorates due to deformation and less susceptibility to the wind. However, the deterioration was about 3 degrees in the range of measurement, so the effect was small.

Thus, it can be concluded that *D*_*L*_ measurements could be made under real-world field conditions with an accuracy of 9.6° of MAE_D_ on average, and it is expected that accuracy will deteriorate at higher wind speeds, with measurements taken with an accuracy of around 12.3°.

#### Accuracy comparison of laboratory and field measurements.

In laboratory experiments, it has been reported that *L*_*w*_ and *C*_*L*_ could be measured within 4% of MAPE and *D*_*L*_ within 6° of MAE [14]. A possible reason for the larger error observed in the current study compared to the reported laboratory experiment is the difference in the reference. In the laboratory experiment, static loads with weights were used, whereas the reference utilized in the current study was based on the six-axis load cell measurements. The six-axis load cell has a certain amount of error, which means that the error in this study may be partially overestimated. In addition, we were unable to determine the cause of the tendency for *C*_*L*_ at 10 Hz to be smaller than the C_Lref_ value when the *C*_*L_ref*_ value is large (Fig 5b, e and h), but it may be due to the characteristics of the load cell used as the standard. The inability to set the pulling azimuth and elevation angle as precisely as in laboratory experiments when conducting pulling tests in the field is also considered to be a cause of the larger errors.

### Advantages of the proposed approach

#### Nondestructive and dynamic measurement.

In this study, we used a load cell to obtain the reference values. It may be reasonable to accept that using a load cell to measure the *L*_*w*_, *C*_*L*_, and *D*_*L*_ values would be sufficient. However, when using a load cell, it is necessary to cut the trunk and fix it on the instrument, which results in a limited measurement period to avoid degradation of the sample quality. The sizes of trees are also limited by the instrument’s measurement capacity, and it is typically impossible to measure trees taller than only a few meters. In contrast, the method employed in the current study can be used by attaching strain gauges to the trunk of a standing tree without cutting it, and, in principle, there is no limit in terms of the size of the individual tree to be measured. The greatest advantage of the employed method is the ability to measure *L*_*w*_, *C*_*L*_, and *D*_*L*_ acting on a standing tree in a nearly nondestructive manner.

In the following, we discuss the applicability of this method to the size of the sample trees. The time resolution to be captured depends on the assumed phenomenon relative to the wind loads acting on the trees. An attempt has been made to estimate quasistatic wind loads as the mechanical loads affecting tree survival, based on the large frequency difference between the wind speed fluctuation and the sway of the tree [21]. However, frequency analyses of tree sway and wind speed fluctuations have indicated that the timing of gust onset coinciding with the sway corresponding to the direction of the gusts is important in terms of evaluating wind-induced tree falls [22]. It has also been observed that the forced swaying caused by a gust with a certain peak frequency in a typhoon caused trees to fall [23]. These findings imply that instantaneous wind loads are important in the evaluation of wind-induced tree falls. Tree sway responds to the fluctuations of wind load, and when measuring dynamic behavior, it is reasonable to focus the analysis on the first-order natural frequency of the sway [24]. To discretize the dynamic behavior of a tree with a first-order natural frequency (f_n_), a sampling frequency (f_s_) must be satisfied under the condition that f_s_ > 2*f_n_ [25]. The maximum f_n_ of the sample trees observed in the current study was 1.5 Hz (Table 1); thus, the sampling frequency of 10 Hz employed in this study was sufficient to capture the dynamic behavior of the sample trees. In addition, the sampling frequency requirement to measure dynamic behavior is relaxed as the tree grows because the f_n_ of a tree decreases with increasing height [26]. Thus, the sampling frequency of 10 Hz employed in this study is valid even when measuring individual trees that are taller than the sample trees considered in this study. However, while the swaying of slender trees such as coniferous trees is governed by a single natural frequency, trees with large branch masses such as broad-leaved trees exhibit more complex swaying[27], so further research is needed to determine whether this method can be applied to trees with complex structures such as broad-leaved trees.

In addition, drag coefficients are frequently used to approximate loads, and gust factors are often used to approximate instantaneous loads. Such methods using coefficients are convenient ways to approximate loads. The method utilized in the current study could also be useful to improve the accuracy of both drag coefficients and gust factors. In this study, we treated the drag coefficient as an indicator of deformation of crown and susceptibility to the wind, but the characteristics of the drag coefficient have been studied in various ways in the past, including its dependence on wind speed, projected canopy area, tree mass, canopy porosity, and the aeration of the canopy [18,19,28,29]. Generally, these characteristics have been investigated in uniform flow using a wind tunnel apparatus. However, the behavior of the drag coefficient in response to dynamic wind load fluctuations has not been fully clarified in field observations, such as the case where a large instantaneous drag coefficient [30] and large fluctuations in the drag coefficient due to tree swaying [17] were observed. The method employed in the current study is effective at elucidating the characteristics of the dynamic drag coefficient.

In terms of the gust factors, the instantaneous loads can be approximated by multiplying the gust factor by the mean loads. However, the gust factor of the wind load varies with the distance from the edge of the forest [31], and the gust factor of wind speed is affected by several factors, including surface roughness and atmospheric stability [32], which make it difficult to obtain accurate values. Using the method employed in this study, it should be possible to identify and characterize the gust factor because the mean and instantaneous load values can be obtained easily.

#### Centroid quantification.

Recently, the response of trees has been analyzed dynamically, including examining the relationship between the structure of tree stem, branches, and leaves and the swaying of individual trees[33,34], and examining the collisions between individual trees caused by swaying and the stability of individual trees[23]. In these studies, the sway was measured using various devices, including prisms [35], inclinometers [21,36,37], extensometers[17], displacement transducers [38], strain gauges [26,39] and accelerometers [40,41]. There have been attempts to estimate the dynamic variation of moments by multiplying the sensor readings by a coefficient that expresses the relationship between the tree motion and the moment [39,42,43]. However, in all of these devices, it is not possible to quantify the wind load and the centroid of wind load separately. In other words, another feature of our method is that it is possible to quantify the moment by separating it into wind load and centroid.

The height of the centroid is frequently given as the centroid of the projected area of the tree crown to the vertical plane [17,44]. This assumes that the wind has uniform velocity pressure across the projected area of the canopy, i.e., a uniform flow is present, and that the drag coefficient is spatially uniform. Thus, the further away the wind speed distribution is from uniform flow, or the more heterogeneous the spatial distribution of the drag coefficient is, the further it is from the centroid of the projected area. This affects the calculation of wind loads and moments acting on the individual tree.

Based on photographs, the centroid of the projected area of the crown to the vertical plane (relative height of the centroid to the tree height) was calculated, and the values for Trees 1–3 were 1.15 m (0.56), 1.55 m (0.66), and 1.64 m (0.57), respectively, and these values were up to 9% greater than *C*_*L*_ and *C*_*L_ref*_ values.

To calculate the moment, *C*_*L*_ and *C*_*L_ref*_ values are multiplied by *L*_*w*_, and the critical wind speed is calculated as the wind speed that balances the moment with the resistive strength of the root system and the bending strength of the trunk [11]. Assuming that the centroid is overestimated by 9%, and *L*_*w*_ is calculated accurately, the critical wind speed is estimated to be 4.2% lower than the true value. Generally, the critical wind speeds range 10–40 m/s [9,11,13,45]. Therefore, the critical wind speed will be underestimated by 0.4–1.7 m/s. In this study, the difference between the centroid from the tree shape and from *C*_*L*_ and *C*_*L_ref*_ was small because there were open areas near the main wind direction, and the vertical wind speed distribution in the canopy was relatively uniform. However, the *C*_*L*_ value may deviate significantly from the centroid of the projected area of the tree canopy when there is a significant vertical distribution of wind speed, and the distribution of branches and leaves is not spatially uniform, such as in a forest environment. Although the centroid is an important factor in terms of estimating the moments acting on the tree, there are few cases where it has been measured. The method employed in the current study could be used to elucidate the relationship between forest conditions and the mechanical processes that convert wind energy into moment.

## Conclusions

Both tree growth and survival are strongly related to wind loads; thus, development of a technique to measure wind loads acting on trees can provide a technical basis to understand tree morphogenesis and forest ecosystems. In this study, the magnitude, direction, and centroid of the wind load distribution acting on trees were measured under natural wind conditions using a previously proposed method [14]. We verified the accuracy of the method regarding wind speed and the variation of wind speed/direction and considered the possibility of applying this method under real-world field conditions. Our findings are summarized as follows:

(i) The wind load (*L*_*w*_) measurements can be performed practically with minor influences of wind turbulence and tree deformation under real-world field conditions, with an accuracy of less than 10% of the systematic errors and the MAPE in the range of higher wind speed of more than 6 m/s.
(ii) The centroid of the wind load distribution (*C*_*L*_) measurements could be made under real-world field conditions with an accuracy of approximately 0.17 m of MAE and 10.8% of MAPE on average; even better accuracy can be expected at higher wind speeds with an accuracy of approximately 0.10 m of MAE and 7.7% of MAPE.
(iii) The wind load direction (*D*_*L*_) measurements could be made under real-world field conditions with an accuracy of 9.6° of MAE on average, but it was predicted that the accuracy would decrease at higher wind speeds with an accuracy of approximately 12.3°.
(iv) The method employed in this study had sufficient characteristics to measure higher standing trees than the current sample (2–3 m of tree height) in terms of sampling frequency.

Thus, the method employed in this study can be widely used to measure dynamic *L*_*w*_, *C*_*L*_, and *D*_*L*_ of standing trees with the above accuracy.

## Supporting information

S1 File The procedures of pulling test and the apparent value of the modulus of elasticity and the radial position of each strain gauge, and the accuracy using determined parameters. (DOCX)
