# Supplementary material for: Measuring the dynamic wind load acting on standing trees in the field without destroying them
Source: PLoS One. 2025 May 20;20(5):e0323532. doi: 10.1371/journal.pone.0323532 (PMC12091786; doi:10.1371/journal.pone.0323532)
Supplement: S1 File — (DOCX) [file pone.0323532.s001.docx]

**The procedures of pulling test and the apparent value of the modulus of elasticity and the radial position of each strain gauge, and the accuracy using determined parameters.**

**1. The procedures of pulling test**

Pulling tests were conducted by manually pulling the trunk horizontally with a string attached at the height of the center of gravity of the sample trees. The applied force was measured using a tension and compression load cell (LUX-B-200N-ID, Kyowa Electronic, Tokyo, Japan) attached to the string. Here, the process of slowly applying and releasing the load was repeated three times, and the maximum applied load was approximately 50 N. The pulling operation was performed sequentially from eight directions at intervals of roughly 45° azimuth, and the direction of the loading was measured using a compass. The applied load was synchronized with strain gauge measurement, and recorded using a data logger (EDX2000A or CR3000X). The data analysis was carried out in the same way as in the previous study [14]; that is, the values obtained from the measurements in eight directions were fitted using a cosine function against the pulling direction. Here, E was determined from the maximum value of the fitting curve, and θ was determined from the phase of the peak of the fitting curve.

2. **Apparent value of the modulus of elasticity and the radial position of each strain gauge**

The *E* and *θ* values thus determined are shown in the table below. Note that the pulling tests were performed when there was no rainfall and nearly no wind.

The determined *E* and *θ* obtained by the pulling test.

**3. Measurement accuracy using determined parameters.**

Measurement accuracy was evaluated by comparing the traction force measured by the load cell with the traction force calculated using *E* and *θ* as determined by the test. As a result, MAPE was 7.6% for *L_w_*, 3.0% for C_L_, and 4.9°for *D_L_*, as shown in the figure below.


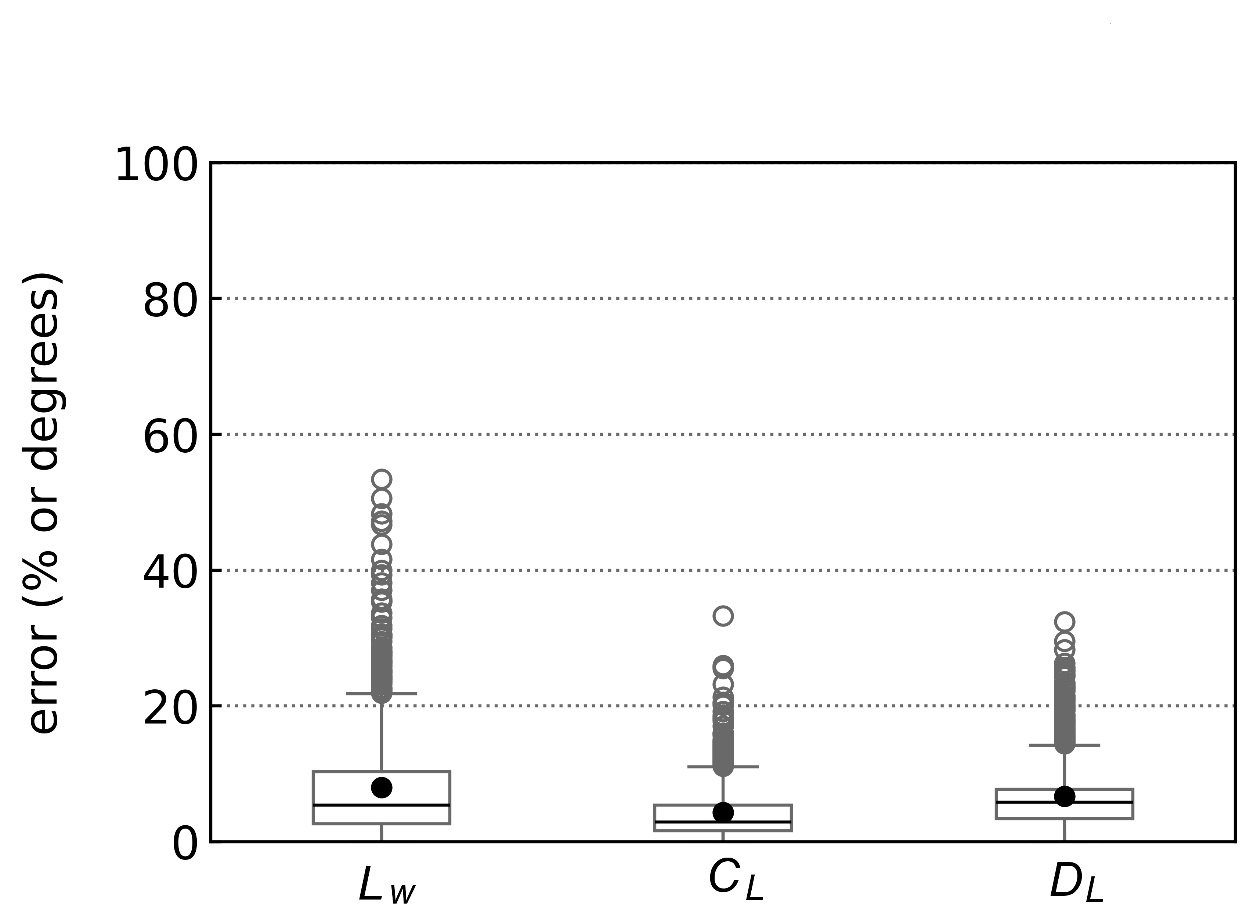


MAPE of *L_w_*, *C_L_*, and *D_L_* between pulling traction measured by a load cell and the value calculated by our method.
